# Effectiveness of technological interventions on psychosocial well‐being and perception of technological interventions among people with Parkinson's disease: A systematic review

**DOI:** 10.1111/ajag.70034

**Published:** 2025-05-02

**Authors:** Terence Kenneth Lau, Man‐Kei Tse, Yaqin Liu, Angela Y. M. Leung

**Affiliations:** ^1^ The Hong Kong Polytechnic University School of Nursing Hung Hom Hong Kong; ^2^ The Hong Kong Polytechnic University WHO Collaborating Centre for Community Health Services Hong Kong China; ^3^ The Chinese University of Hong Kong Hong Kong China; ^4^ The Hong Kong Polytechnic University Research Institute for Smart Ageing (RISA) Hong Kong China

**Keywords:** digital health, Parkinson's disease, psychological well‐being

## Abstract

**Objectives:**

The increasing number of technological interventions related to Parkinson's disease (PD) signifies growing research interest in the PD technological domain. It remains unknown how these interventions could affect the psychosocial health of people with PD. This systematic review aims to explore how technological interventions affect people with PD psychosocial well‐being and their perception towards these interventions.

**Method:**

A systematic review was conducted using Cochrane Library®, Embase®, IEEE Xplore Digital Library®, PsycInfo®, PubMed® and Web of Science® databases following PRISMA guidelines. Two individual assessors conducted quality appraisals using the Mixed Method Appraisal Tool. Both quantitative narrative and qualitative thematic synthesis were adopted to analyse the extracted data.

**Results:**

This review included 27 articles with 752 people with PD, with seven categories of technologies implemented in physical rehabilitation. Qualitative findings indicated the overarching theme of coping with technological intervention. Three themes were identified: user perception of intervention design and functional appropriateness, attitude shift and coping, and perceived benefits from technological interventions. Unsuccessful coping attempts and overcomplicated intervention designs induced negative emotions and affected the psychosocial well‐being of people with PD.

**Conclusions:**

Although most PD technological interventions focused on physical rehabilitation, people with PD reported a psychosocial gain in improved autonomy and reinforced social relationships during the intervention period. A better rewardability intervention design was considered more satisfying and could promote self‐acceptance rather than stress‐inducing. Interventions' technological complexity should match participants' expectations and technological literacy to facilitate the coping process with the intervention for people with PD. More research would be required to quantify the reported psychosocial gain and examine the technological literacy of people with PD when designing a more appropriate intervention regime.

**Practice impact**

The current review identified the potential psychosocial benefits of implementing technological physical rehabilitative interventions among PD populations. To support the adaptation of the interventions, professional supports and introductory sessions could be provided, and involving people with PD to co‐design and develop the interventions could also be considered.

## 1 INTRODUCTION

The prevalence of Parkinson's disease (PD) has doubled to 6.1 million individuals worldwide in recent years.^1^ People with PD not only experience a wide range of motor and non‐motor symptoms but also comorbidity with psychiatric disorders.^2^ They experience worsening psychosocial symptoms along their disease progression, which could lead to reduced social activities and social seclusion.^3^ The self‐reported loneliness of people with PD not only documented how it affected their depressive and sleep dysfunction symptoms' severity,^3^, ^4^ but also how it could induce self‐stigmatisation, a persistent obstacle in PD history.^5^, ^6^

The construct of psychosocial well‐being often overlaps with the quality of life (QoL) construct, as the World Health Organization (WHO) defined the latter as a multi‐dimensional construct that spanned across physical, psychological and social domains in how an individual would evaluate their position in life based on their goals, expectations and concerns.^7^, ^8^ Ambiguity often leads to varied definitions across the literature^9^ when QoL is considered synonymous with subjective well‐being^10^ or subjective well‐being with additional human needs.^11^ Well‐being involves psychological, social and emotional well‐being domains, as established in the early 90s.^12^, ^13^, ^14^ It includes an individual's optimal functioning on personal and societal levels, emphasising positive effects and avoiding undesirable emotions^15^ and could serve as a protective factor for both motor and non‐motor PD symptoms.^16^

Conventional non‐pharmacological interventions supporting people with PD primarily focus on physical rehabilitation^17^ to slow down PD progression or as symptom relief. With recent technological advancements, technology elements have been imbued within PD research for multidisciplinary telemedicine, wearable remote sensor monitoring, virtual reality (VR) gait training, exergaming in physical therapy or exoskeletons and robotic gait assistance for neurorehabilitation.^18^ These elements could benefit the physical and cognitive functions among people with PD who are over 65 years old.^19^ A meta‐analysis revealed that the combination of Nintendo Wii and physiotherapy was more effective in promoting balance and QoL than traditional rehabilitation alone.^20^ Other reviews identified different technological applications, such as wearable devices^21^, ^22^ and VR rehabilitation^23^ that could also promote QoL and enhance physical attributes, such as stride length, gait and balance, without noticeable adverse effects.

While the efficacy of technological intervention on the physical attributes of people with PD has been extensively researched in technological PD interventions, their other focal point, QoL, unavoidably overlaps with the physical domain, leading to an inconclusive efficacy within the psychosocial domain. In addition, with only one review that briefly examined the usability of VR training for stroke patients and people with PD,^24^ how people with PD perceive these technological interventions and their efficacy within psychosocial well‐being remains unknown in the literature. This review aimed to explore how technological interventions affect the psychosocial well‐being of people with PD and how people with PD feel about these interventions.

## 2 METHODS

### 2.1 Literature search

Five databases were selected for the current review, namely Embase, MEDLINE, Web of Science, PsycInfo and CENTRAL from Cochrane Library with e‐publications ahead of their printings. These databases were systematically searched in November 2021 using the Population, Intervention, Comparison and Outcome (PICO) search strategy. Additional Medical Subject Headings (MeSH) for PubMed were used. Search keywords and their synonyms were shown in Table 1.

**TABLE 1:** Search keywords and strategy for databases and MeSH term for PubMed.

Categories	Keywords and synonyms
Population (P)	
Target population AND	aged [MeSH] OR aged[Title/Abstract] OR older adults[Title/Abstract] OR Senior[Title/Abstract] OR over age 60[Title/Abstract] OR over age 65[Title/Abstract] OR elderly[Title/Abstract] OR Young‐old [Title/Abstract] OR old‐old[Title/Abstract] OR long‐term care[Title/Abstract] OR late life[Title/Abstract] OR old age[Title/Abstract] OR older people [Title/Abstract]
Condition	Parkinson Disease[MeSH] OR Parkinson Disease[Title/Abstract] OR parkinsonism [Title/Abstract] OR parkinson's disease[Title/Abstract] OR Parkinson[Title/Abstract] OR parkinsonian syndromes [Title/Abstract]
Intervention (I)	Wearable Electronic Devices [MeSH] OR wearable technology[Title/Abstract] OR wearable sensors[Title/Abstract] OR wearable[Title/Abstract] OR technology[Title/Abstract] OR technologies[Title/Abstract] OR technological intervention[Title/Abstract] OR Robotics[Title/Abstract] OR Robotic[Title/Abstract] OR Robots[Title/Abstract] OR Robot [Title/Abstract] OR social robots[Title/Abstract] OR surgical robots[Title/Abstract] OR assistive robots[Title/Abstract] OR Robotic system[Title/Abstract] OR Virtual reality[Title/Abstract] OR VR[Title/Abstract] OR Augmented reality[Title/Abstract] OR AR[Title/Abstract]
Outcome (O)	psychosocial well‐being[Title/Abstract] OR psychosocial wellbeing[Title/Abstract] OR psychological well‐being[Title/Abstract] OR psychological wellbeing[Title/Abstract] OR Subjective well‐being[Title/Abstract] OR Subjective wellbeing[Title/Abstract] OR social well‐being[Title/Abstract] OR social wellbeing[Title/Abstract] OR Life satisfaction[Title/Abstract] OR Happiness[Title/Abstract] OR Quality of life[Title/Abstract] OR social connectedness[Title/Abstract]
Combination	P AND I AND O

### 2.2 Screening and eligibility criteria

Articles were required to fit the following inclusion criteria in the current review: (1) have people with PD as their target population; (2) quantitative, qualitative or mixed‐method study design; (3) primary research with technological intervention; (4) involves at least one method to explore user perception or psychosocial well‐being of people with PD; (5) available full‐text; (6) published between 2000 and 2022. The current review focused on research after 2000 since the technological PD research trend has been steadily growing since the late 2000s, and more were published after 2016.^25^

Articles were excluded if the following criteria were met: (1) involved technological intervention that solely acted as a platform for professionals to provide therapy; (2) involved in medication titration; (3) brain stimulation studies; (4) grey literature, such as conference papers, presentations or posters; (5) not written in English; and (6) study protocol.

### 2.3 Data extraction

The title and abstract of each article were screened by the researcher, followed by a subsequent full‐text screening. A manual search included three articles with embedded qualitative elements^26^, ^27^, ^28^ from the key journal (*Sensor*) and Google Scholar since the user perception related to the psychosocial well‐being domains of people with PD was not explicitly stated in the abstract. Data were extracted from the eligible literature for further analysis: (1) the basic information (first author, publishing year and location), (2) study design, (3) demographic data of participants, (4) details of intervention (dosage, duration and frequency), (5) related psychosocial domains and (6) result.

### 2.4 Quality appraisal

A Mixed Method Appraisal Tool (MMAT version 2018)^29^ was used to evaluate the quality of the selected paper. The MMAT is considered a unique and promising tool^30^ that allows investigators to appraise quantitative, qualitative and mixed‐method studies using the same appraisal tool. The MMAT recorded a moderately replicable to perfect consensus inter‐rater reliability in its previous version. Two reviewers (L.T.K. and T.M,K) appraised the quality of each article independently based on the following procedures. Researchers completed the two screening questions and then categorised articles into three main categories: qualitative, mixed‐method and quantitative study design to appraise their respective criteria.^31^

Quality assessment identified a good fit between research designs and questions. The MMAT rating ranged from 25% to 100% (25% for each criterion passed). Twenty‐five studies passed more than three assessment criteria. One study that scored 25% was a case study of a PD patient, hence the relatively high risk of bias. Eight studies utilised various qualitative research elements, from open‐ended questions within the questionnaire to individual semi‐structured interviews with purposeful sampling. Only three studies were classified as mixed‐method or qualitative studies according to the standards of the MMAT. All inter‐rater discrepancies were discussed until consensus was reached, and no paper was excluded due to low‐quality ratings.^29^

### 2.5 Data analysis

A mixed‐method data synthesis approach was adopted to clarify how complex interventions could serve the PD population.^32^ A thematic synthesis approach proposed by Thomas and Harden^33^ was adopted to synthesise the appropriateness, acceptability and perceived effectiveness from extracted qualitative data and which aimed for conceptual saturation. The process started with inductive free coding on selected articles, without hierarchical structure, in order to understand the phenomenon without the constraint of the research objectives. Similar codes were then grouped for descriptive themes and revisited for consistency and interpretation. Finally, analytical themes were synthesised based on the research objectives that were established on the primary data, but also with a fresh perspective and understanding of the reviewed topic.^34^

With most of the included articles (*n* = 24) having integrated QoL measurements, eight QoL and five different emotional well‐being measurements were implemented to assess the subjective well‐being domains of people with PD. Meta‐analysis was not feasible for synthesising quantitative data due to the wide variety of implemented psychometric tools. Therefore, due to high heterogeneity, a narrative synthesis was adopted to analyse quantitative data.^35^

Ethics statement is not applicable for the current review as it only involves published journal articles.

## 3 RESULTS

**FIGURE 1:**
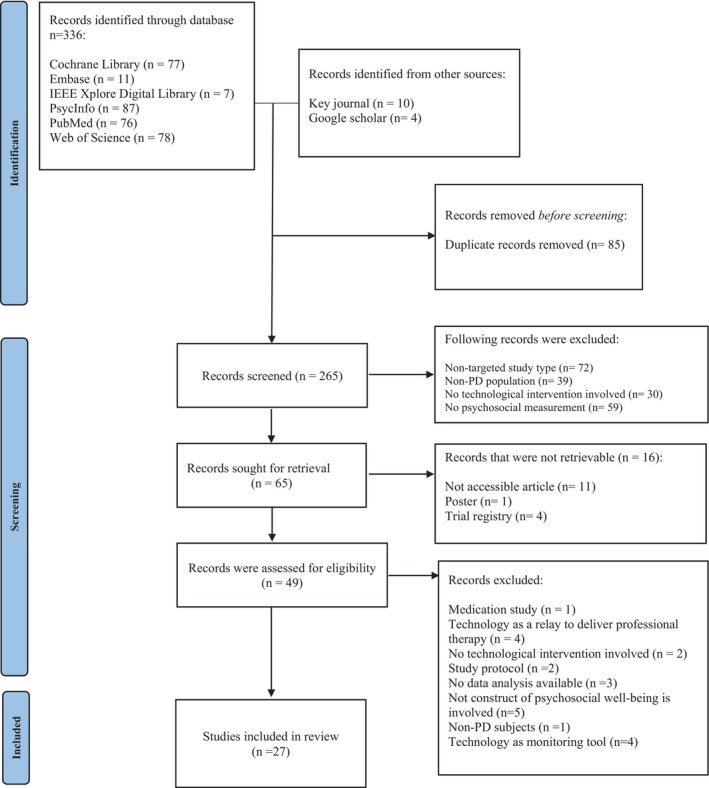
PRISMA flow chart.

The systematic search of six databases retrieved 336 publications. A further 14 papers were included from the key journal (*Sensor*) and Google Scholar. After removing duplications, 265 studies' abstracts and titles were screened for eligibility. Forty‐nine journals were successfully extracted for full‐text review, and 27 studies fulfilled the inclusion criteria. The screening process is illustrated with the PRISMA flow chart^36^ shown in Figure 1.

### 3.1 Overview of reviewed articles

Twenty‐seven studies across 13 countries published from 2010 to 2022 were selected for this review. There were 24 quantitative studies, two qualitative studies and one mixed‐method study, with 12 being pilot studies or case reports. The majority of the reviewed articles were published from 2018 onward (*n* = 19), and the interventions lasted an average of 6.4 weeks. About half of the studies were completed in Europe (*n* = 13), while a few studies were done in North America (*n* = 5), Asia (*n* = 5) and South America (*n* = 4). Different technologies were used in these studies: gaming applications (*n* = 8); gait and balance training (*n* = 8); wearable devices (*n* = 4); mobile applications (*n* = 3); telehealth system (*n* = 2); metronome (*n* = 1); social robot (*n* = 1); and VR/AR technology (*n* = 11). Extracted data are presented in Table 2.

**TABLE 2:** Psychosocial‐related user perception and effectiveness of PD technological intervention.

Authors, years, place and setting	Study design	Sample	Intervention	Related well‐being domains	Perception	Effectiveness
Wearable device						
Cochen De Cock et al.^48^ France Setting: Community—Home based	Quantitative non‐RCT with pre–post design	Total participants: 45 Mean age: 65 Male: 56% MDS‐UPDRS‐III: ≥1 and <3	Smartphone application and a wearable sensor (BeatWalk) for gait rehabilitation by modifying music tempo to induce spontaneous gait Duration: 4 weeks Frequency: 5 days/week	SubWB‐ Positive emotion ‐Measured at T0: Baseline T1: Week 4	More neutral responses were noted on user enjoyment	EQ‐5D: QoL score significantly improved (mean score: 9.14 at T0; 7.86 at T1, *p* = 0.05) LARS: and degree of apathy was significantly decreased (mean score: ‐7.3 at T0; ‐9.0 at T1. *p* = 0.02) BDI and PAS: No significant differences (*p* = 0.91 & 0.57 respectively)
Tunur et al.^42^ USA Setting: Community	Quantitative non‐RCT with pre–post design	Total participants: 7 Mean age: 69 Male: 43% H and Y stage: >3	AR dancing application (moving through glass) with google glass to improve mobility and balance Duration: 3 weeks Frequency: Daily	SubWB‐ Positive and negative emotion ‐Measured at T0: Baseline T1: Week 3	Participants would regard using the application as an enjoyable experience and identified positive effects from intervention Barriers identified as confusing instructions, difficult controls and uncomfortable to wear	PDQL and BDI: No significant differences (statistic not shown)
Hermanns et al.^40^ USA Setting: Community	Quantitative descriptive with pre–post design	Total participants: 5 Mean age: 73 Male: 60% H and Y stage: 1–4	The combination of a physical activity tracker (Fitbit Alta HR) and a tablet (iPad) to view exercise videos and access online support group Duration: 12 weeks Frequency: Three times/week	SocWB‐ Social integration PWB‐ Positive relations with other ‐Measured at T0: Baseline T1: Week 12	Reported with negative evaluation, such as ‘terrible’, ‘do not like it’ or ‘never very good at it’ if people with Parkinson's disease fail to master the operation techniques Themes identified: *Encouragement* Messages shared were inspiring, courage and hope inducing among peers *Support* This theme was most prominent in regards on using a tracker and doing physical activity, thinking of a workaround themselves *Group participations* If all participants were more involved, it could enhance the likability of the intervention Frustration was noted if people with Parkinson's disease cannot participate in such social interaction	FACT‐G: No significant difference identified in total score and the following subscales (Wilcoxon signed‐rank tests. *p* > 0.05) Physical well‐being Social/ Family well‐being Emotional well‐being Functional well‐being
Volpe et al.^54^ Canada Setting: Clinical	Quantitative RCT with pre–post and follow‐up design	Total participants: 40 G1 Mean age: 66.5 G2 Mean age: 69.6 Male: 40% H and Y stage: ≥2	Wearable Proprioceptive Stabiliser (EquistasiH) with combination of physiotherapy for rehabilitation of postural instability Duration: 2 months Frequency of wearing the intervention: 1–3 weeks: 6 days/week 4–8 weeks: 5 days/week G1: active device G2: Inactive device	No WB‐related subdomain scores were reported from PDQ‐39 ‐Measured at T0: Baseline T1: Weeks 8–9 T2: Week 16		PDQ‐39: G1 has a significant improvement on QoL score from T0–T1 (‐48.8%, *p* = 0.004) and improvement retained at T2 in G1 (*p* = 0.039)
Mobile apps						
Kim et al.^49^ Korea Setting: Community	Quantitative non‐RCT with pre–post design	Total participants: 21 Mean age: 72.4 Male: 19% H and Y stage: 1–4	A mobile application to facilitate exercise, including stretching, aerobic, balance and coordination and vocal exercises Duration: 8 weeks Frequency: Duration set by therapist	SubWB‐ Satisfaction Negative emotion ‐Measured at T0: Baseline T1: Week 8	Positive responses on self‐reported usability questionnaire, such as ‘satisfaction’, ‘role expectation for rehabilitation’ and ‘adequate difficulty’ were noted from participants	GDS—short form: Significant improvements (*p* = 0.04) in depression PDQ‐39: Significant improvements in total score (*p* = 0.02), but non‐significant change in emotional well‐being domain (*p* = 0.93)
Campo‐Prieto et al.^26^ Spain Setting: Community	Qualitative study	Total participants: 4 Mean age: 62.2 Male: 75% H and Y stage: 2	An immersive virtual environment accessed by HTC Vive ProTM for physical rehabilitation Duration: Two sessions in 2 weeks Frequency: Not specified	SubWB‐ Satisfaction	GEQ scores highlighted positive items Positive response from Satisfaction Ad Hoc Questionnaire includes the intervention being entertaining and fun, realistic motivating and relaxing One respondent replied that is their best experience to date while some other also claim the intervention period was too short	
Ginis et al.^55^ Belgium Setting: Community	Quantitative RCT with pre–post and follow‐up design	Total participants: 40 Mean age: Not specified Male: Not specified H and Y stage: 2–3	CuPiD system, a smartphone device with two built in applications, ABF‐gait (audio biofeedback) and FOG‐cue (instrumented cueing) G1: CuPiD training G2: Control training Duration: 6 weeks Frequency: Three times/week	EWB‐ Balance of positive and negative emotions ‐Measured at T0: Baseline T1: Week 6 T2: Week 10		SF‐36: Reported with significant time by group effect (F_(2,108)_ = 1.58, *p* < 0.05, η^2^ = 0.06) on physical health domain. QoL maintained in intervention group but noted deterioration for control among people with Parkinson's disease No significant difference on mental health subdomain score for both groups (*p* = 0.75)
Gaming						
van Beek et al.^39^ Switzerland Setting: Community	Quantitative descriptive with pre–post design	Total participants: 10 Mean age: 65.4 Male: 70% H and Y stages: 2–4	Exergaming (Leap Motion Controller) Dexterity Intervention with augmented VR Duration: 4 weeks Frequency: Two time/week	SubWB‐ Positive emotion ‐Measured at T0: Baseline T1: Week 4	Participants were noted with generally positive impressions and enjoyed the interactive elements of the intervention, but a high level of concentration was required because of intervention's difficult level The low sensitivity of the device was reported by people with Parkinson's disease and two of them wished the intervention to last longer	PDQ‐39: Significant time × group interaction effect in total (F_(1,8)_ = 8.53, *p* = 0.02, η^2^ = 0.52)score for poor dexterity participants, while no significant improvement could be found with good dexterity participants (*p* = 0.93) No subjective well‐being/ social subdomain scores of PDQ‐39 was reported
Sánchez‐Herrera‐Baeza et al.^27^ Spain Setting: Community	Mixed methods	Total participants: 6 Mean age: 74.5 Male: 83% H and Y stage: 1–4	Serious gaming (immersive VR) with leap motion to improve the function of upper extremities Duration: 6 weeks Frequency: Three times/week	PWB‐ Personal growth Positive relations with other Autonomy SocWB‐ Social integration SubWB‐ Negative emotions Satisfaction	Psychological: Participant feels awkward, nervous, at fear and surprised during the adaptation period, but gradually becoming more comfortable participants believed the intervention is more of a mental challenge than a physical one, it improved their mental and cognitive state more than the targeted physical state Social: The intervention encouraged participants to share their experience among themselves, provide mutual support and facing the intervention together Barriers and facilitators: Overcoming challenges would give them a sense of satisfaction, become more competent on daily living and bring them closer to their family. But failed to do so would lead to sense of frustration Nervousness and tension were noted as they wish to do their best. Boredom experienced was originated from the monotonous intervention design	Participants showed a high degree of satisfaction with CSQ‐8. (mean of 3.66/4)
Nuic et al.^41^ France Setting: Not specified	Quantitative non‐RCT with pre–post and follow‐up design	Total participants: 10 (with FOG and/or postural instability) Mean age: 64.2 Male: 50% H and Y stage: ≥3	A customised videogame hosted in Kinect system, where participants would response to visual/auditory cues Duration: 9 weeks Frequency: Three sessions/week	SubWB‐ Positive emotion ‐Measured at T0: Baseline T1: 9th session T2: 18th session T3: Week 12	Seven out of 10 participants rated the game as amusing	EPN‐31: Non‐significant change (χ^2^(5) = 3.08, *p* = 0.69) identified in participants' negative affect, with their positive affects decreasing over time (F_(5,45)_ = 1.88, *p*‐0.11) PDQ‐39SI: Non‐significant change in QoL (*p* = 0.13) No subjective well‐being/ social subdomain scores of PDQ‐39SI was reported
Alves et al.^47^ Brazil Setting: Laboratories	Quantitative non‐RCT with pre–post and follow‐up design	Total participants: 27 Mean age: 61 Male: 93% H and Y stage: 1	Motor and cognitive games that hosted on Xbox Kinect and Nintendo Wii G1: Nintendo Wii™ G2: Xbox Kinect™ G3: Control group (no rehabilitation) Duration: 5 weeks Frequency: Two sessions/week	SubWB‐ Negative emotion ‐Measured at T0: Baseline T1: Week 1 T2: Week 5		BAI: G1 were noted with significantly decreased anxiety levels from T0‐T1 (F_(2,19)_ = 3.76, *p* = 0.045) and sustained till follow‐up (*p* = 0.031) G2 failed to improve anxiety level (p≥ 0.05, power= 0.112) WHOQOL‐OLD: No significant difference on QoL among all three groups (*p* ≥ 0.05, power = 0.102)
Ferraz et al.^56^ Brazil Setting: Community	Quantitative RCT with pre–post design	Total participants: 62 Mean age: 69 Male: 60% H and Y stage: 2–3	Exergaming with Xbox 360 Kinect, containing a series of mini games Duration: 8 weeks Frequency: Three sessions/week G1: Functional training G2: Bicycle exercise, G3: Exergaming Goal: Walking capacity	SubWB‐ Negative emotion ‐Measured at T0: Baseline T1: Week 8		GDS: No significant difference across all groups (*p* > 0.05) G1: EQ‐5D: Significantly improved total score (*p* = 0.014) PDQ‐39: No significant difference (*p* = 0.069) G3: EQ‐5D: No significant difference (*p* = 0.31) PDQ‐39: Significantly improved total score (*p* = 0.004) No subjective well‐being/ social subdomain scores of PDQ‐39 was reported
Pompeu et al.^57^ Brazil Setting: Community	Quantitative non‐RCT with pre–post design	Total participants: 7 Mean age: 72 Male: 86% H and Y Stage: 2–3	Using Kinect games for motor and cognitive training, includes elements of balancing and postural adjustments Duration: 6 weeks Frequency: Three sessions/week	No WB‐related subdomain scores were reported from PDQ‐39 ‐Measured at T0: Baseline T1: Week 6		PDQ‐39: Improved total mean score ( T0= 27.8 T1 = 22.34 )
Santos et al.^58^ Brazil Setting: Community	Quantitative RCT with pre–post design	Total participants: 45 Mean age: 64.3 Male: 76% H and Y Stage: 1–3	Virtual rehabilitation (VR) with Nintendo Wii Duration: 8 weeks Frequency: Two sessions/week G1: Nintendo Wii G2: Conventional Exercises G3: Nintendo Wii + Conventional Exercises	No WB‐related subdomain scores were reported from PDQ‐39 ‐Measured at T0: Baseline T1: Week 8		PDQ‐39: Significantly improved QoL among all groups (*p* < 0.05). But no significant between group difference (*p* = 0.331)
Yuan et al.^58^ Taiwan Setting: Clinical	Quantitative RCT with pre–post and follow‐up design	Total participants: 24 G1 Mean age: 67.8 G2 Mean age: 66.5 Male: 46% H and Y stages: 1–3	Customised interactive video game‐based (IVGB) training (XaviX entertainment system) on balance Duration: 12 weeks Frequency: 3 days/week Gp1: 6‐week intervention + 6‐week control phase (no exercise) Gp2: 6‐week control phase + 6‐week intervention	SubWB‐ SF‐36 ‐Measured at T0: Baseline T1: Week 6 T2: Week 12		SF‐36: No significant difference observed between all subscales (*p* > 0.02) after adjusting alpha value to .0031 with Bonferroni method
Telehealth						
Chatto et al.^38^ Georgia	Quantitative descriptive with pre–post design	Participant: 67 years old Female H and Y Stage: 2 Setting: Community	System for Technology‐Augmented Rehabilitation and Training (START) incorporated with an AI assistant that would provide guidance and feedback to participants when they were doing home exercise program Duration: 4 months Frequency: N.A.	PWB‐ Self‐acceptance SubWB‐ Satisfaction ‐Measured at T0: Baseline T1: Week 4 T2: Week 16	Noted with high satisfaction on intervention system and it is easy to learn, operate and timing saving nature, praising its reliability and privacy Reported being pleased with her overall experience but would be discouraged from her perceived poor performance and being confused with the feedback mechanism during the semi‐structured interview	PDQ‐39: Scores decreased slightly from 30 to 25 (T1 and T2), indicating an improvement in her overall quality of life from T0 No subjective well‐being/ social subdomain scores of PDQ‐39 was reported
Isernia et al.^43^ Italy Setting: Community	Quantitative non‐RCT with pre–post and follow‐up design	Total participants: 62 Mean age: 66.8 Male: 54.8 H and Y stage: = <2	The Human Empowerment Aging and Disability (HEAD) tele‐rehab program (VR) contain videos to improve balance, strength of upper and lower extremities and cognitive aspect, such as memory and dual‐task capabilities Duration (clinic): 1 month Frequency (clinic): three times/week Duration (home): 1 months Frequency (home): 5 times/week G1: HEAD Program G2: Usual Care	SubWB‐ Positive and negative emotions ‐Measured at T0: Baseline T1: Week 4 (end of ClinicHead) T2: Week 16 (end of HomeHead) T3: Week 28		SF‐12: Significantly improved mental health subscale from T0 to T1 (t = 2.18, df = 29, *p* = 0.029 ) PANAS: Significant improvement on positive affect from T0 to T1 (t = 2.35, df = 30, *p* = 0.019)
Training						
Brandín‐De la Cruz et al.^60^ Spain	Quantitative non‐RCT with pre–post design	Total participants: 12 Mean age: 68.8 Male: 58% H and Y stage: Not specified Setting: Clinical—inpatient	Immersive VR and antigravity treadmill training for gait rehabilitation Duration: 4 weeks Frequency: Three sessions/week	SubWB‐Satisfaction SF‐36 ‐Measured at T0: Baseline T1: Week 4	7/9 participants reported being ‘very satisfied’ with the training on a 5‐point Likert scale, while the others rated the intervention as ‘quite satisfied’	SF‐36: Significant differences associated with small‐medium effect sizes on physical functioning (*p* = 0.027, d = 0.4), role physical (*p* = 0.049, d = 0.6), and bodily pain (*p* = 0.018, d = 0.5). But no significant change in mental health (*p* = 0.212, d = 0.4) and social functioning (*p* = 0.263, d = 0.4) subscale
Capecci et al.^61^ Italy	Quantitative RCT with pre–post design	Total participants: 110 Mean age: 67.6 Male: 45% H and Y stage: ≥2 Setting: Community	Robot‐assisted gait training (G‐EO system) for gait rehabilitation Duration: 4 weeks Frequency: 5 days/week G1: Robot‐assisted gait training G2: Treadmill training	No WB‐related subdomain scores were reported from PDQ‐39 ‐Measured at T0: Baseline T1: Week 4		PDQ‐39: Significant time effect (F = 41.9, *p* < 0.0001), but non‐significant group by time effect (F = 1.6, *p* = 0.2)
Lo et al.^45^ USA Setting: Community	Quantitative non‐RCT with pre–post design	Total participants: 4 with FOG Mean age: 63.3 Male: 75% H and Y stage: Not specified	Robot‐assisted treadmill (exoskeleton) training to reduce FOG episodes and improving gait Duration: 5 weeks Frequency: Two sessions/week	SubWB SocWB ‐Measured at T0: Baseline T1: Week 5		PDQ‐39: No meaningful changes in Communication subscale Meaningful effect size changes on Emotional well‐being (d = ‐0.56) and Social Support (d = ‐0.52)domains. These improvements were described as ‘unexpected’ by authors
Paker et al.^62^ Turkey Setting: Community	Quantitative non‐RCT with pre–post and follow‐up design	Total participants: 12 Mean age: 65.5 Male: 50% H and Y stage: 1–3	Robotic treadmill training on the functional mobility and walking capacity in the ambulatory participants Duration: 5 weeks Frequency: Two session/week	No WB‐related subdomain scores were reported from PDQ‐39 ‐Measured at T0: Baseline T1: Week 5 T2: Week 12		PDQ: Total score was significantly improved at T1 (*p* = 0.03), while the improvement did not sustain to week T2 (*p* = 0.0117) HADS: Only measured at baseline
Wang et al.^63^ China Setting: Clinical—inpatient	Quantitative non‐RCT with pre–post and follow‐up design	Total participants: 52 G1 Mean age: 60.1 G2: Mean age: 62 Male: 56% H and Y stage: 1–3	C‐Mill (augmented reality treadmill) gait training Goal: To improves walking adaptability Duration: 1 week Frequency: Daily G1: Postural instability/gait difficulty group G2: Non postural instability/gait difficulty group	No WB‐related subdomain scores were reported from PDQL ‐Measured at T0: Baseline T1: Week 1 T2: Week 12		PDQL: Only G2 reported with significant improvement (*p* = 0.021), no between group difference could be identified (*p* = 0.378)
Yang et al.^64^ Taiwan Setting: Community—Home based	Quantitative RCT with pre–post and follow‐up design	Total participants: 23 G1 Mean age: 72.5 G2 Mean age: 75.4 Male: 74% H and Y stages: 2–3	VR balance training system Duration: 6 weeks Frequency: Two session/week G1: VR balance training G2: Conventional training	No WB‐related subdomain scores were reported from PDQ‐39 ‐Measured at T0: Baseline T1: Week 6 T2: Week 8		PDQ‐39: Significant improvement on total score for both groups and retained till T2 (T1 < T0, *p* = 0.047; T2 < T0, *p* = 0.022) No significant group by time effect could be identified (*p* = 0.806).
Pilleri et al.^65^ Italy Setting: Laboratory	Quantitative non‐RCT with pre–post design	Total participants: 20 Median age: 64.5 Male: Not specified H and Y stage: 2.5–4	An over‐ground motor driven footboards that would move at a stable speed, and can be adjusted according to participants' ability Duration: 3 weeks Frequency: 5 days/week	No WB‐related subdomain scores were reported from PDQ‐8 ‐Measured at T0: Baseline T1: Week 3		PDQ‐8: Significantly improved total score (*p* = 0.033)
Pazzaglia et al.^44^ Italy Setting: Community	Quantitative RCT with pre–post study design	Total participants: 51 Mean age: 71 Male: 69% H and Y stage: Not specified	VR rehabilitation, with Each VR session consisted of multiple exercises Duration: 6 weeks Frequency: Three sessions/week G1: Virtual reality program G2: Conventional Program	SubWB‐ Balance of positive and negative emotions ‐Measured at T0: Baseline T1: Week 6		SF‐36: Significant improvement on the mental composite score in G1 (*p* = 0.037)
Robot						
Wilson et al.^28^ USA	Qualitative study	Total participants: 10 Mean age: Not specified Male: Not specified Stage: Early to moderate Setting: Not specified	Social robot with medication sorting abilities Duration: One time Frequency: N.A.	SubWB‐ Negative emotion	Generally reported with negative reactions; for example, of frustration, confusion and struggle when operating the robot Considered using social robot for medication sorting is an overkill Annoyance was noted from the lack of assistance provided	
Other						
Elston et al.^46^ UK	Quantitative RCT with cross‐over and ‘wash‐out’ period design	Total participants: 42 Mean age: 71.5 Male: 67% H and Y stage: 2–4 Setting: Community	Portable electronic metronome as acoustic cueing to improve mobility and ADL Duration: 10 weeks Frequency: N.A. G1: Early group G2: Late group	SubWB ‐Measured at T0: Baseline T1: Week 4 T2: Week 10 T3: Week 14		SF‐36: Subjective meaningful change identified in role limitation (emotion) but non‐significant group by time interaction on all subdomains (*p* > 0.25) PDQ‐39: Non‐significant group by time interaction across all psychosocial domains (*p* > 0.1) and reduced QOL in the emotion domain (score increased by 0.83)

Abbreviations: BAI, Beck Anxiety Inventory; BDI, Depression Inventory; CSQ‐8, Client Satisfaction Questionnaire; EPN‐31, Positive and Negative Emotionality Questionnaire; EQ‐5D, EuroQol‐5 Dimension; FACT‐G, functional assessment of cancer therapy—general; GDS, Geriatric Depression Scale; GEQ, Game Experience Questionnaire (postgame module); H&Y, Hoehn & Yahr; HADS, Hospital Anxiety and Depression Scale; LARS, Lille Apathy Rating Scale; PANAS, positive affect and negative affect schedule; PAS, Parkinson Anxiety Scale; PDQ, Parkinson's Disease Questionnaire; PDQL, Parkinson's Disease Quality of Life Questionnaire; PWB, psychological well‐being; SF, Short Form Health Survey; SocWB, social well‐being; SubWB, subjective well‐being; WHOQOLOLD, World Health Organization Quality of Life for Older Persons.

The 27 studies involved 752 participants, with a sample size ranging from 1 to 110 and a mean participant count of 28. The mean age of people with PD ranged from 61 to 74.5 years old, and 50% of participants were male. Twenty‐two studies adopted the Hoehn and Yahr staging scale^66^ to assess the motor functioning of their participants.

### 3.2 Thematic synthesis of qualitative data

‘Coping with PD technological interventions’ was identified as the overarching theme of the current review. Three psychosocial well‐being‐related themes were identified throughout the coping process: user perception of intervention design and functional appropriateness, attitude shift during coping attempts and the perceived psychosocial benefits from technological interventions.

### 3.3 Users' perception of the intervention design and functional appropriateness

Functional appropriateness represents the ability of an intervention to execute its intended usage.^37^ Before people with PD come into contact with the technological interventions, they will form an expectation of its intended use, design and duration. An appropriately designed intervention could induce a sense of satisfaction or reduce negative emotions when using underachieved interventions.

Intervention design and module duration were of concern among people with PD. An overcomplicated design led to a sense of frustration, struggle and annoyance. People with PD stated that using a social robot for medication sorting was ‘overkill’ when a smartphone would be considered ‘sufficient’ for medicine management.^28^ On the operational aspect, people with PD were confused by the feedback mechanism should it lack sufficient elaboration or provide contradictory audio and visual performance evaluations within an AI‐assisted home exercise program.^38^ On the contrary, if the intervention presented high reliability, high privacy and was easy to learn and operate, people with PD would be pleased with their overall experience and rate the intervention with a high satisfaction rating.^38^

Intervention duration was explored in exergaming studies by Campo‐Prieto et al.^26^ and van Beek et al.^39^ where intervention duration was set between 10 and 30 min per session. Ten out of 13 participants were satisfied with the session duration, and the remaining indicated a preference for an extended session, showing that participants appreciated appropriately designed interventions.

### 3.4 Attitude shift during coping attempts

#### 3.4.1 Initial emotional responses

Undesirable emotions were shown in people with PD upon the initial contact with the technological interventions when they failed to achieve their intended use. Poor performance evaluation in task‐based interventions was reported to cause people with PD nervousness, tension and discouragement, as participants wanted to excel in the intervention study or achieve a higher score.^27^, ^38^ In addition, frustration was reported in the activity tracking study by Hermann et al.^40^ if participants failed to engage in online social interaction via the embedded online support group. Awkwardness, fear, boredom and frustration were also reported from people with PD when they tried a series of VR gaming interventions.^27^ In contrast, pleasant feelings were also identified, where people with PD found interventions amusing and enjoyable in gaming.^39^, ^41^ and AR dancing.^42^

#### 3.4.2 Barriers that were encountered

Technological difficulties encountered by people with PD were focused on system operation, levels of difficulties and cognitive demand. People with PD found it challenging to navigate the intervention system if the system had poor hardware sensitivity or confusing instructions, and feedback was given. Technical support was required from fellow participants and researchers for possible workarounds/guidance.^28^, ^38^, ^39^, ^40^, ^42^

In addition, people with PD regarded VR gaming interventions as more cognitively demanding than the intended physical challenges and were more of a mental challenge than physical rehabilitation.^27^ Similar mental challenges were observed in the exergaming intervention, where participants claimed the intervention required a high concentration level due to its difficulty setting.^39^

#### 3.4.3 Attitude shift

If people with PD successfully overcame the aforementioned challenges and transitioned through the initial adaptation period, the reported nervousness and awkward feelings gradually subsided as they became comfortable and experienced in managing the intervention.^27^ Similar scenarios were also found in the activity tracking study,^40^ where people with PD initially evaluated the intervention negatively when they failed to cope with the intervention (through iPad) but had no problems working through it once they figured it out.

### 3.5 Perceived psychosocial benefits from technological interventions

#### 3.5.1 Social support

Improved peer interactions and relationships were identified from the reviewed articles. People with PD experienced a sense of satisfaction and were closer to their family after overcoming the previously mentioned technological and cognitive challenges.^27^ The activity tracking study by Hermann et al. encouraged people with PD to share their experience of performing physical activities with fellow participants in their virtual support group.^40^ Prosocial behaviours were expressed through peer encouragement and support. These peer interactions were described as inspiring, hope‐inducing and supportive.

During the VR gaming study, mutual support among participants was observed by the sharing of their experiences of overcoming challenges and comparing scores with each other.^27^ Such interactive features between the exergaming intervention and people with PD were highly valued.^39^

#### 3.5.2 Autonomy

From the VR gaming intervention,^27^ people with PD asserted that the intervention had more effect on mental and cognitive state than physical attributes, and it promoted their autonomy by helping them become more competent in daily living.

### 3.6 Effectiveness on improving psychosocial well‐being

#### 3.6.1 Emotional well‐being

The positive and negative emotional feelings of people with PD were the most quantitatively measured psychosocial domain among the reviewed articles and were often embedded into QoL scales. Among the 24 studies measuring QoL, 14 utilised the PD Questionnaire (PDQ; *n* = 14), and six adopted the Short Form‐12 and 36 (SF‐12 and SF‐36). While only nine (four RCTs; four non‐RCTs; one descriptive study) reported the psychosocial subscale of the QoL scale, three (one RCT and two non‐RCTs) documented the following significant subdomain changes (see Table 3).

**TABLE 3:** Psychosocial improvements identified from reviewed articles.

Reviewed articles	Study type	Scales adopted	Effectiveness
Wearable device			
Cochen De Cock et al.^48^	Quantitative non‐randomised	EQ5‐D LARS	Overall QoL score significantly improved Degree of apathy was significantly decreased
Volpe et al.^54^	Quantitative RCT	PDQ‐39:	Significant improvement on overall QoL score
Mobile apps			
Kim et al.^49^	Quantitative non‐randomised	GDS—short form PDQ‐39	Significantly reduced in depressive symptoms Significantly improved total score, but not emotional well‐being subdomain
Ginis et al.^55^	Quantitative RCT	SF‐36	Significant time by group effect on physical health domain, but no significant difference on mental health subdomain
Gaming			
van Beek et al.^39^	Quantitative descriptive	PDQ‐39	Significant time × group interaction effect in total score for poor dexterity participants
Alves et al.^47^	Quantitative non‐randomised	BAI	Significantly reduced anxiety levels in Nintendo Wii™ group till follow‐up
Ferraz et al.^56^	Quantitative RCT	EQ‐5D PDQ‐39	Functional training group: Significantly improved total score Non‐significant changes Exergaming group: Non‐significant changes Significantly improved total score
Pompeu et al.^57^	Quantitative non‐randomised	PDQ‐39	Improved total score
Santos et al.^58^	Quantitative RCT	PDQ‐39	Significantly improved QOL across all groups
Telehealth			
Chatto et al.^38^	Quantitative descriptive	PDQ‐39	Improved QOL score on one participant
Isernia et al.^43^	Quantitative non‐randomised	SF‐12 PANAS	Significantly improved mental health subscale Significantly improved positive affect
Training			
Brandín‐De la Cruz et al.^60^	Quantitative non‐randomised	SF‐36	Significant differences documented in physical domains, but not mental health subscale
Capecci et al.^61^	Quantitative RCT	PDQ‐39	Total score decreased by 15%
Lo et al.^45^	Quantitative non‐randomised	PDQ‐39	Meaningful effect size changes on Emotional well‐being and Social Support domains, but not in Communication subscale
Paker et al.^62^	Quantitative non‐randomised	PDQ	Significantly improved overall score
Wang et al.^63^	Quantitative non‐randomised	PDQL	Only non‐postural instability/ gait difficulty group reported with significant improvement
Yang et al.^64^	Quantitative RCT	PDQ‐39	Significantly improved total score for both groups with pairwise comparisons
Pilleri et al.^65^	Quantitative non‐randomised	PDQ‐8	Significantly improved total score
Pazzaglia et al.^44^	Quantitative RCT	SF‐36	Significantly improved mental composite score
Metronome			
Elston et al.^46^	Quantitative RCT	SF‐36	Subjective meaningful change in role limitation (emotion)

Abbreviations: BAI, Beck Anxiety Inventory; EQ‐5D, EuroQol‐5 Dimension; GDS, Geriatric Depression Scale; LARS, Lille Apathy Rating Scale; PANAS, positive affect and negative affect schedule; PDQ, Parkinson's Disease Questionnaire; PDQL, Parkinson's Disease Quality of Life Questionnaire; SF, Short Form Health Survey.

The VR rehabilitation conducted by Isernia et al.^43^ and Pazzaglia et al.^44^ significantly improved mental composite scores in the SF‐12 and SF‐36, respectively. It was suggested that VR rehabilitation could be integrated with telerehabilitation and retain its beneficial effect (although the interventions usually had a small effect size) until follow‐up^43^ and was considered more effective than the conventional regime^44^ In addition to the significant QoL improvement recorded by Isernia et al.,^43^ they also identified a significant correlation between participants' positive mood and intervention adherence and a small effect size on the Positive Affect and Negative Affect Scale.

Other meaningful changes in QoL were documented in treadmill training,^45^ where a small‐to‐moderate effect size (−.49 to −.56) was reported across the PDQ‐39's emotional and social subdomains among four participants. The authors further noted that such psychosocial subdomain improvements were unexpected. Although there was no significant difference between the SF‐36 and PDQ‐39 scores in a metronome RCT study,^46^ the reported subjective clinically improved SF‐36 emotion (role limitation) domain suggested possible mental health benefits that warrant a larger scale RCT for confirmation.

Among the other eight reviewed studies measuring depressive and anxiety symptoms in people with PD, four studies recorded significant mood improvements after using the interventions. A significant reduction of anxiety symptoms was observed in the motor and cognitive gaming intervention, and such effect was sustained for 1 month after the five‐week intervention.^47^ This effect was only observed within the Nintendo Wii but not in the Xbox Kinect group. Another wearable intervention in a gait rehabilitation study, BeatWalk, significantly reduced the apathy level (via the Lille Apathy Rating Scale) of people with PD, but not Beck's Depression Inventory nor the Parkinson Anxiety Scale score.^48^ Finally, a pilot mhealth exercise study identified a reduction in depressive symptoms in the Geriatric Depression Scale—short form.^49^

#### 3.6.2 Social well‐being

Social well‐being improvements were also highlighted in the reviewed qualitative data. Two studies explored the perks of encouraged social interaction among people with PD.^27^, ^40^ The integration of social interaction induced a sense of unity and mutual support among people with PD and their families, through face‐to‐face sharing^27^ or via online support groups.^40^

## 4 DISCUSSION

Almost all the reviewed studies (26 out of 27) focused on physical rehabilitation, while psychosocial well‐being was not set as the focus of the investigation. Developing and implementing interventions that aim to improve the psychosocial well‐being of people with PD has not yet been fully explored, but both quantitative and qualitative data showed promising results of using technological intervention to promote psychosocial well‐being among people with PD, even if they were focused on physical rehabilitation.

### 4.1 Unexpected effect on psychosocial well‐being

Quantitatively speaking, there was limited data about psychosocial well‐being among people with PD. Although the current review was able to identify potential therapeutic effects from technological interventions, they predominately targeted physical rehabilitation and measured emotional well‐being within QoL scales or a few other psychometric scales on emotions. There is a lack of quantitative evidence on the effect of these technological interventions on the psychological and social well‐being domains of people with PD. Therefore, no conclusive statement could be drawn on their therapeutic impact on the psychosocial well‐being of people with PD. Future psychosocial research among the PD population could capitalise on this research gap in designing psychosocial‐oriented technological PD interventions and address this lack of unified psychosocial measurements by adopting psychosocial‐orientated scales, such as the Mental Health Continuum Short Form,^14^ to explore and quantify their interventions' psychosocial effectiveness comprehensively.

Secondly, the reviewed qualitative data complemented the existing quantitative psychosocial improvements by capturing the attitude shift of people with PD during the adaptation period. In addition, the unexpected psychosocial improvements in this review echoed another systematic review, which suggested multidisciplinary physical rehabilitation therapy targeting mobility and activities of daily living can significantly reduce depressive and anxiety symptoms among people with PD.^2^

This social well‐being promoting nature also aligns with a recent review reporting interactive social elements in technological interventions that could benefit community‐dwelling older adults.^67^ At the same time, more evidence is required to compare the effectiveness of online and offline communication modes on social well‐being among people with PD.

In addition, a VR rehabilitation study^27^ highlighted the psychological well‐being gained from its participants. Participants reported their autonomy would be promoted alongside improved performance in activities of daily living, and the intervention would improve their mental and cognitive state more than the intended physical attributes, echoing the extracted quantitative data. The authors from the robot‐assisted treadmill training described the PDQ‐39 mental and social subscale improvements as unexpected.^45^ However, without reporting the applied psychosocial‐related QoL subdomains, previous studies missed the opportunity to quantify the reported psychosocial gain from technological interventions, making it impossible to make a conclusive statement on the effectiveness of these interventions on psychosocial well‐being.

### 4.2 Factors affecting the psychosocial well‐being of people with Parkinson's disease

#### 4.2.1 Coping with intervention

The coping process with technological intervention has been identified as the psychosocial well‐being determining factor. A recent systematic review conducted by Sevcenko and Lindgren^24^ briefly mentioned that an unsuccessful implementation of VR physical training among stroke and PD older adults could have originated from participants' negative preconceptions. This review resonated with their findings and further delineates that undesirable emotions developed during the adaptation period could transition into a more positive side upon successful coping. Whether an intervention could be successfully implemented should not be solely based on the preconception but on participants' technological literacy, available technical support and intervention design.

#### 4.2.2 Technological literacy

Despite the rapid technological advancement in PD research, the lack of intervention on the technological illiteracy of people with PD has been stressed among scholars^50^ and stays true among selected articles. Technological literacy has been described as the ability of an individual to operate, manage, assess and make sense of a given technology and is categorised into awareness, praxis (training and practices) and phronesis (technological competence).^51^ An individual has to be aware of and familiar with the use and functionalities of the technology to become competent.^52^

Participants who experienced technical difficulties in the reviewed articles displayed a lack of computing knowledge and the necessary skills to operate the interventions. They remained at the first two levels of technological literacy (awareness and praxis), requiring technical support from others due to not being aware of the latest technological trends or not being familiarised with the system navigation. For example, people with PD who reportedly grew up using punch cards instead of computers and were unfamiliar with the implemented smart tablet^40^ or had difficulties comprehending the instructions for operating social robots and Google Glass.^28^, ^42^ Negative psychosocial influences, such as frustration, struggle and annoyance, would persist without appropriate intervention.^28^

Some people with PD were noted to exhibit phronesis levels of technological literacy in some rare incidents and took the advisory role of guiding their peers in need.^40^ Technology‐literate individuals were believed to be more comfortable and objective with technology, resulting in fewer negative emotions when interacting with technological products.^51^ Noteworthy, such actions can simultaneously facilitate peer support and promote social well‐being among people with PD.

The gap between the technological literacy of people with PD and the technological demand of the implemented intervention could contribute to a prolonged adaptation, undesirable emotions and a reduced possibility of people with PD mastering the intervention. Future research should therefore provide sufficient technical support from research staff, or adopt ‘training the trainer’ to promote peer support among people with PD and enhance the capability and technological literacy among people with PD in navigating the intervention.

#### 4.2.3 Intervention rewardability and design

The complexity and rewardability of the intervention would also affect the psychological well‐being of people with PD. Good intervention performance would induce a sense of achievement and encourage self‐acceptance, and the improved activities of daily living could also grant them a sense of autonomy.^27^, ^38^ Some task‐based interventions were considered by people with PD to be stress‐inducing, resulting in undesirable emotions or discouragement due to poor performance.^27^, ^38^

The extracted user reviews also generated insight into designing a more PD‐friendly intervention. People with PD considered having professional supervision, setting introductory sessions,^27^, ^39^ and a more progressive module duration according to the ability of people with PD^42^ should help reduce the adaptation period. By gradually increasing the module duration as the subject progresses, researchers can create a task‐based intervention with fitting difficulties, maintaining an appropriate intervention difficulty. Interventions should be challenging enough to achieve the intended physical rehabilitation goal and induce a sense of achievement instead of discouraging people with PD. Future research could take in recommendations from people with PD, or consider adopting the co‐design approach by involving people with PD in the intervention development, improving the existing practices, maximising the intended therapeutic outcomes,^53^ and empowering the involved people with PD.

### 4.3 Limitations

#### 4.3.1 Quality of selected studies

Due to the heterogeneity of the reviewed papers, the current review adopted a narrative synthesis but not a meta‐analysis approach. Therefore, no effect size can be calculated. In addition, due to the relatively small sample size (44% of the reviewed articles were pilot studies, *n* = 12) and limited amount of the data presented, there is a possibility that the current review cannot fully capture the psychosocial effect of the selected technological interventions.

Although qualitative data were extracted and synthesised into reported themes, the level of evidence was of low quality. Thus, a rigorous qualitative methodological approach was not adopted and such studies cannot be classified as mixed‐method design by the standards of the MMAT, leading to reduced trustworthiness for qualitative data. More sophisticated qualitative methodology and empirical studies (e.g. large‐scale RCT) would be required to examine the effectiveness of PD technological intervention.

## 5 CONCLUSIONS

This systematic review identified the rising trend in exploring how technological intervention could interfere with the psychological well‐being of people with PD, summarised the users' perception of using technological interventions, and examined the potential effects of these interventions on the psychosocial well‐being of people with PD.

We identified *coping with the technological intervention* as an overarching theme, where the user perception of intervention design and functional appropriateness, attitude shift during coping attempts and the perceived psychosocial benefits from technological interventions were linked to the psychosocial well‐being of people with PD. Technological interventions specific to PD have the capability of enhancing the psychosocial well‐being of people with PD and are worth further investigation. The technological literacy of people with PD, and intervention design could be considered before implementing technological interventions in order to shorten the adaptation period and reduce undesirable emotions among people with PD.

## CONFLICT OF INTEREST STATEMENT

No conflicts of interest declared.

## Data Availability

The data that support the findings of this study are available from the corresponding author upon reasonable request.
